# Effect of Barrel-to-Barrel Variation on Color and Phenolic Composition of a Red Wine

**DOI:** 10.3390/foods10071669

**Published:** 2021-07-20

**Authors:** Leonard Pfahl, Sofia Catarino, Natacha Fontes, António Graça, Jorge Ricardo-da-Silva

**Affiliations:** 1 LEAF—Linking Landscape, Environment, Agriculture and Food Research Center, Instituto Superior de Agronomia, Universidade de Lisboa, Tapada da Ajuda, 1349-017 Lisboa, Portugal; leonard.pfahl@dlr.rlp.de (L.P.); jricardosil@isa.ulisboa.pt (J.R.-d.-S.); 2 CeFEMA—Center of Physics and Engineering of Advanced Materials, Instituto Superior Técnico, Universidade de Lisboa, Av. Rovisco Pais, 1, 1049-001 Lisboa, Portugal; 3 Sogrape Vinhos S.A., 4430-809 Avintes, Portugal; natacha.fontes@sogrape.pt (N.F.); antonio.graca@sogrape.pt (A.G.)

**Keywords:** red wine, oak barrel aging, cooperage, barrel-to-barrel variation, phenolic composition

## Abstract

Tangible variation of sensory characteristics is often observed in wine aged in similar barrels. Barrel-to-barrel variation in barrel-aged wines was investigated in respect of the most important phenolic compounds of oenological interest. A red wine was aged in 49 medium-toasted oak (*Quercus petraea*) barrels, from four cooperages, for 12 months. The resulting wines were evaluated for chromatic characteristics, anthocyanin-related parameters, total phenols, flavonoids and non-flavonoids phenols, flavanol monomers, and oligomeric and polymeric proanthocyanidins. PCA and ANOVA were applied to investigate the relationships between barrels and to assess cooperage and individual barrel effect. Three cooperages influenced the wine similarly during aging. Anthocyanin-related parameters showed the highest variation, 25–37%, other phenolics varied 3–8.5%, and with two exceptions, chromatic characteristics changed 1.7–3%. The relationship between the number of barrels and the expected variation for each analytical parameter was calculated, as reference for future measurements involving barrel lots, either in wine production or experimental design.

## 1. Introduction

Wine aging in oak barrels is a traditional and widespread practice in winemaking worldwide. Alternative containers such as stainless-steel tanks, concrete vessels, or polyethylene tanks surpass barrels in some respects, such as price, hygiene, and material homogeneity. Nevertheless, barrels are still firmly established in quality wine production and that is due to their positive influence on the organoleptic quality and complexity of wine [1]. Various phenomena related to physical and chemical characteristics of the oak are directly responsible for these effects. First, the water and ethanol non-negligible evaporation due to the porosity of the wood [2] and some wine absorption by the wood (especially in new barrels). Second, the transfer of extractable compounds such as ellagitannins and volatile substances such as guaiacol, eugenol, and ethyl- and vinyl phenols as well as oak lactones (β-methyl-γ-octalactone) and furfural (-derivates) [3]. The total amount though is limited and quickly reduced by the extraction process into wine [4]. The extracted substances influence sensations such as astringency and mouthfeel and increasing the aroma intensity and complexity. Third, the oxygen moderate permeation and diffusion through the wood promotes different reactions of oxidation, polymerization, copigmentation, and condensation involving anthocyanins and proanthocyanins which stabilize the color and reduce the astringency. Finally, the storage in barrels accelerates the natural sedimentation of unstable colloidal matter, thus contributing to wine stability and limpidity [1].

Phenolic changes during wine aging are a dynamic process yielding a huge variety of colorless products and pigments [5]. Their relative quantity depends on many factors [6]. Some of these changes have their origin in oxygenation. In wine, the component highest in quantity after water is ethanol, leading to acetaldehyde being a main product of wine oxidation. Acetaldehyde, furfural, hydroxymethyl furfural and glyoxylic acid react according to the Friedel–Crafts reaction mechanism with flavanols and hydrated forms of anthocyanins [7]. Traces of acetaldehyde are protonated in an acidic environment producing powerful electrophiles which further react with wine nucleophiles. The reactions with different flavonoids leading to polymerization reactions are desired, as they are not very stable and can rearrange into stable pigments, specifically pyranoanthocyanins and xanthylium [8]. Acetaldehyde reacts as well directly with anthocyanins as for example with malvidin-3-glycoside to yield vitisin B [9]. Vitisins are resistant to bleaching by bisulphite and contribute to stable wine color [10]. Oxygen availability and the anthocyanin to flavanol ratio are regulating factors to these reactions [5]. When in the carbinol base form, anthocyanins act like a nucleophile and are able to take part in many reactions with flavanols [6,11] as well as polymerization reactions with o-quinones [12]. The concentration of anthocyanins therefore decreases with wine aging as they react with various molecules to what is conventionally summarized under the term polymeric pigments. Important polymeric pigments are pyranoanthocyanins: vitisins, methylpyranoanthocyanins, vinylphenolic pyranoanthocyanins, pinotins, and portisins [5]. Wine color is stabilized not only by the formation of polymeric pigments but as well by copigmentation, which describes the association of anthocyanins with other compounds leading to a spectral shift in color [5,13]. No covalent bonds are formed. In young wines 30–50% of the color is due to copigmentation because of the high percentage of anthocyanin monomers, which would without copigmentation be present in their colorless carbinol base form [13].

Proanthocyanidins undergo under acidic conditions spontaneous cleavage of their interflavanic bonds [14]. This leads to a constant bonding and bond breaking, resulting in both the formation of bigger and smaller molecules [12,14]. The reaction with anthocyanins seems to inhibit further polymerization and therefore leads to oligomeric rather than polymeric molecules which is assumed to reduce astringency [5,15]. The chain length of polymerized proanthocyanidins can be expressed in the mean degree of polymerization. Polymeric tannin contributes to wine perception as with increasing molecular weight, also wine astringency increases [16]. 

Barrel is made from the natural product wood. The most commonly used species are *Quercus petraea* (sessiliflora oak), *Quercus robur* (pedunculated oak) and their hybrids and *Quercus alba* (white American oak). Locally, alternative botanical species, other than oak may be used as well [17]. The wood composition as well as the production process underlie a variation [18]. The main influencing factors are the oak species and origin of wood [19,20], the seasoning and its location [21], and the toasting process in the cooperage [4,22]. Barrels influence wine phenolic composition and color development during aging. For this reason, the phenolic compounds are likely to be affected by barrel-to-barrel variation. In fact, this variation is also widely known to winemakers resulting in tastings and analytical control of individual barrels. Despite these facts there is little to be found concerning barrel-to-barrel variation in the literature. 

Towey and Waterhouse [23] studied the variation of seven volatile phenols deriving from a Chardonnay wine fermented in lots of each 10 barrels of four different cooperages. Reported is an average variance of 27% in a range from 15% to 40% for the volatile phenols, leading to more than 50% of variation for a single barrel experiment. For the results of an analysis to stay within a 20% range of the population mean, on average 10 barrels per barrel lot were required. However, the content of extractable volatile phenols depends mainly on the toasting process, therefore, the general influence of a barrel on wine phenolics and color is likely to be different. A recent study of Pilet et al. [24] found no statistical difference for the variation of trace elements from barrel to barrel. However, variation of flavonoid content was reported as well as the differentiation of color intensity, tonality, total anthocyanins, ionized anthocyanins, total pigments, and polymerized pigments for one-barrel type. 

The variation of barrel influence can be problematic for scientific studies which include barrels in their experimental design. Up to this date there is little knowledge of to what extend results might be affected by natural barrel variation although bearing the potential of result misinterpretation. The problem gains importance with smaller sample batches, which due to economic reasons are often the case for experimental designs including barrels. 

This study aims to shed light on the variating influence of barrels on wine color, pigments, and phenolic composition of wood-aged wine. The trial stands out due to its practical background with a wine produced at industrial scale. The high number of 49 barrel samples of four cooperages result in a high robustness of the results. To our best knowledge, little information is available in the literature about the variation effect of barrels. The present study therefore represents a unique experimental design which extends the current knowledge.

## 2. Materials and Methods

### 2.1. Wine, Wooden Barrels, and Sampling

A red wine of the grape variety Touriga Franca (*Vitis vinifera* L.), 2017 vintage, from the Portuguese PDO Douro, produced at industrial scale in a real winery, was used in this experiment. The wine resulted from the alcoholic fermentation of must in the presence of the solid parts of the berry (skins and seeds), by application of the conventional red winemaking, at 28 °C. Diammonium phosphate was added in doses of 15 g/hL at the beginning, after one third and after two thirds of the alcoholic fermentation. No pre or post fermentation maceration was performed. The general physical–chemical characteristics of the wine after spontaneous malolactic fermentation, and before being filled in the barrels were as follows: alcoholic strength, 13.3% vol; total dry matter, 27.3 g/L; total acidity, 5.5 g/L (expressed in tartaric acid); volatile acidity, 0.57 g/L (expressed in acetic acid); pH 3.78. The wine was used to fill a total of 49 new French oak (*Quercus petraea*) barrels (225 L) of four different cooperages: A—20 barrels, B—8 barrels, C—9 barrels and D—12 barrels, all with medium toasting level. According to the cooperage, typical drying time of oak wood was 18–24 months and toasting protocols use temperatures from 160 to 200 °C (wood surface) for a few tens of minutes. The barrels were opened during barrel aging twice per month, first for sampling and later for SO_2_ addition and topping up if needed. The SO_2_ corrections were carried out to keep a free SO_2_ level of 40 mg/L, combined with topping up the barrels with wine of the same batch stored in a stainless-steel tank. The storage temperature of the barrels was 15–18 °C while the cellar humidity ranged between 75 and 85%.

At the beginning of the experiment, a sample of the wine was taken in a glass bottle of 0.375 L and sealed with a screw cap, before filling the barrels (November, 2017). This sample was coded as bottle matured wine. Samples of each individual barrel were taken, in glass flasks of 100 mL, after the 12 months of aging.

### 2.2. General Physical and Chemical Analysis

General physical–chemical analysis was carried out by Fourier transform—infrared spectrometry (FOSS Winescan FT-120 FTIR wine spectrometer) [25] for: Alcoholic strength, density, total dry matter, reducing substances, total acidity, volatile acidity, SO_2_, pH, ash, sulfates, and chloride, always in duplicate.

### 2.3. Color, Pigments, and Phenolic Composition

Regarding chromatic characteristics, pigments, and phenolic composition, all analyses were carried out on centrifuged wines (20,000× *g*, 30 min). Absorbance measurements were recorded on a UV-VIS spectrophotometer (Agilent, Santa Clara, CA, USA, Cary 100 UV-Vis). For each measurement, triplicates were performed. 

#### 2.3.1. Chromatic Characteristics

The basic chromatic characteristics color intensity and tonality were measured following the official OIV method [26]. Color intensity is calculated by summarizing the optical densities calculated for 1 cm optical path and the absorbance at 420, 520, and 620 nm wavelength. Tonality is defined as the ratio of absorbance at 420 nm to 520 nm. CIELab parameters (H*, L*, C*, a* and b*) were determined following recommendations of the Commission Internationale de L’Eclairage26 in a 1 mm path length cuvette with a measurement every 5 nm along the 380 to 780 nm wine spectrum.

#### 2.3.2. Total Anthocyanins, Degree of Ionization of Anthocyanins, Ionized Anthocyanins, Total Pigments, Polymerization Index, Polymerized Pigments

The analytical parameters were analyzed following the methodology based on Somers and Evans [27]. The basis of the principle of the method is that by excessive addition of SO_2_ all anthocyanins are decolorized and residual color is due to other sources, such as polymeric pigments. Furthermore, at a pH < 1 all anthocyanins are present in their colored flavylium form, while polymeric pigments are less affected by the low pH. For the calculation degree of ionization of anthocyanins and ionized anthocyanins an arbitrary factor necessary to respect the dependency of polymeric pigments on the pH, was chosen (1.35).

#### 2.3.3. Total Phenols, Flavonoid Phenols, Non-Flavonoid Phenols

Total phenols were determined according to the method established by Ribéreau-Gayon, measuring the absorbance at 280 nm [28]. Non-flavonoids were determined according to the method developed by Kramling and Singleton [29]. The principle of the method is based on the precipitation of flavonoids due to the addition of formaldehyde at low pH. The phenol content is measured after the precipitation and just before by the total phenol analysis. Absorbance was measured at 280 nm wavelength. Flavonoids were then calculated by subtracting non-flavonoids from total phenols.

#### 2.3.4. Tanning Power

Tanning power describes the potential astringency of the wine. It was measured following the method of De Freitas and Mateus [30]. The principle of the method is that tannin, true to the origin of its name, can denaturalize proteins. This protein-polyphenol interaction can be analyzed by nephelometry due to the ability of polyphenols to bind proteins, in this case bovine serum albumin.

#### 2.3.5. Color Due to Copigmentation

This characteristic was assessed according to the method presented by Boulton [13]. Two analyses are performed. In the first acetaldehyde is added to wine with the intention to release anthocyanins combined with SO_2_. In the second analysis a part of the previous sample is diluted in hydroalcoholic solution to dissociate the copigmented anthocyanin complexes. Absorbance is read for both analyses at 520 nm.

#### 2.3.6. Flavanol Monomers and Proanthocyanidin According to Polymerization Degree 

The separation and analysis of wine flavanol monomers, oligomeric, and polymeric proanthocyanidins, was conducted according to the method of Sun et al. [31,32]. Based on the degree of polymerization (DP), flavanol monomers and proanthocyanidins were separated by C18 Sep-Pak cartridges (Waters: Sep-Pak Plus Short-TC18 and Sep-Pack Plus-TC18). Absorbance is measured at 500 nm during a vanillin reaction in an acidic medium.

### 2.4. Statistical Analysis

Statistical analysis was performed with the software R^®^ (version 4.0.0, the R Foundation for Statistical Computing). Data were compiled with Microsoft Excel^®^ 2016 (Microsoft Inc., Redmond, WA, USA). A principal component analysis (PCA) was performed to investigate the relationships of the barrels.

To analyze the effect of the cooperage on the analytical parameters the following strategy was carried out: The data were first tested for normal distribution using the Shapiro–Wilk test. If normally distributed, a Bartlett test followed to analyze the variance homogeneity, if not normally distributed a Levene test was used instead [33]. If data were normally distributed and showed homogenic variance, an ANOVA was carried out to check for significant differences between the four cooperages, if positive a Tukey HSD was used as a post hoc test. Not normal distributed, but variance homogeny data were further analyzed with a Kruskal–Wallis test and if positive with the Dunn test as a post hoc test. Not homogeny data were analyzed with a Brown and Forsythe ANOVA and if positive further analyzed with the DTK test and the Alexander and Govern test. In the post hoc test the factor was the cooperage and the variable the analytical parameter of interest. Cooperages were identified as different when showing a *p*-value below 0.05. 

To identify the variation from barrel to barrel and in between the cooperages, the coefficient of variation (CV) was calculated for each cooperage and analytical parameter by dividing the standard deviation by the mean and is expressed in %. It shows the degree of variability in relation to the population average [33]. Furthermore, the sampling replicates were used to identify the variation associated with the analytical method. For each barrel, the CV of replicate results was determined and in a second step all CVs were averaged for the corresponding parameter.

The practical background and the sample size of the dataset allow calculation of the required barrel number to retain results which lie within a predefined acceptable range between the sample mean and population mean. The following equation was used [34]:

r = ((zα + zβ) ∗ σ/d)^2^

where r is the sample size to be calculated. zα is in this case 1.96, which is the z-value for a normal distribution at 95% confidence level, or the chance of making a type I error of 5%. zβ includes the probability of making a type II error and was set on 20%, meaning the calculation has a power of 80%. The corresponding value is 0.84. The standard deviation of the population is represented by σ. Finally, d implements a predefined variation which is acceptable for the experiment. This is calculated as a part of the population mean: Population mean (μ) divided by the chosen variation, 2%, 5%, 10%, 15%, or 20%. This method follows the example of Towey et al. [23,35]. The equation was used to determine the required barrel number for each combination of parameter, cooperage, and variation level. The required barrel numbers of each cooperage were averaged to gain the average required barrel number for the entire trial. 

## 3. Results and Discussion

The presentation of results and their discussion contains of three subsections of which the first one aims to present the results of the many parameters analyzed from a wine aging perspective. Differences between the four cooperages are pointed out already but the variation in between and within the cooperages is topic of subsection two. In subsection three the effect of this variation on the required barrel number for analytical procedures is explored.

### 3.1. Wine Aging

**Figure 1:**
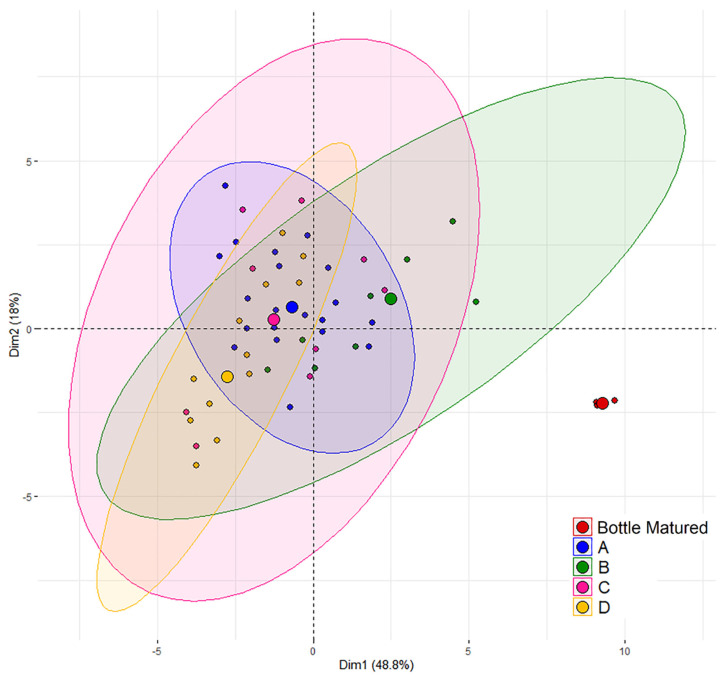
Principal Component Analysis performed on wines aged in oak barrels from the cooperages A, B, C, and D and bottle matured wine, in a total of 50 wines. The wines are represented in the plane of the two first components which express, respectively, 49% and 18% of the total variance.

As aforementioned, the bottle matured wine was collected before barrel filling and kept under reductive conditions, until all samples were analyzed at the same time. However, the comparison of one bottled wine with 49 barrels comes with the drawback regarding a hypothetical variation of bottle aging not being taken into account. The following comparisons need to be seen in this context. The Principal Components Analysis (PCA) (Figure 1) revealed a clear separation of the bottle matured wine from the clustering barrel-aged wines. The variables which influence the separation of the bottle matured wine to the barrel matured wine the most are these describing the color of the wine and wine pigments. Despite this separation in the PCA, no significant differences could be found between the bottle matured wine and the barrel-aged wine for total anthocyanins, degree of ionization of anthocyanins, ionized anthocyanins, polymerization index, color due to copigmentation (%), oligomeric and polymeric proanthocyanidins, as well as total condensed tannins.

#### 3.1.1. General Physical–Chemical Parameters

Alcoholic strength, total dry matter and ash were found to be slightly increased by 3%, 9% and 7%, respectively, in the barrel matured wine compared with the bottle matured wine (Table 1). A possible explanation could be the loss of water and alcohols due to evaporation through the pores of oak wood, occurring when the humidity in the barrel environment is too low and in a minor extent the direct extraction of wood constituents to wine [2]. In respect to total acidity, the average concentration observed in barrel-aged wines was similar to the bottle matured wine. The pH was found to be the same for the bottle matured wine and the barrel-aged wine (Table 1), and lower than that of wine before aging, probably as a consequence of natural tartaric stabilization [36]. Regarding sulfates, the higher concentrations observed in barrel-aged wines compared to the bottle matured wine, on average plus 158%, are most probably explained by the periodically addition of SO_2_ to keep 40 mg/L of free SO_2_. This is necessary as free SO_2_ is constantly declining during barrel maturation as it is oxidized to SO_4_ [1].

#### 3.1.2. Pigments and Phenolic Composition

In comparison to the bottle matured wine, except for non-flavonoids and total anthocyanins, all phenolic parameters showed higher values in the barrel-aged wines (Table 2). Total phenols, flavonoids and tanning power increased by 4%, 10% and 13%, respectively, in the 12 months barrel matured wine compared to the bottle matured wine. For polymerization index, proanthocyanidins fractions and flavanol monomers average increases from the bottle matured wine to the barrel-aged wine were observed, but without statistical significance for the individual cooperages (Table 2). The missing statistical significance is most probably partly explained by the observed analytical method variation in the present trial (Table 3).

In the literature, a slow decrease of phenolic compounds concentrations [16,37] during wine aging is reported as a result of precipitation and adsorption [38,39] as well as a reduction in the degree of polymerization and astringency [16,37,40]. Based on that, the barrel-aged wine was expected to show a lower tanning power and phenolic compounds concentrations in comparison to the bottle matured wine. It is possible that the 12 months aging period was not enough to show these trends. Gambuti et al. [41] compared barrel matured wines and did not find a clear trend for total phenols after the wine maturation period. The increases recorded in this experiment might be explained by the extraction of substances from oak wood to wine as ellagitannins extracted from oak wood might have contributed to the stabilization of phenolic compounds and prevented their precipitation [39]. Non-flavonoids and total anthocyanin decreased by 41% and 16% respectively, in the barrel-aged wine compared to the bottle matured (Table 2). The decrease of non-flavonoids can be explained by the micro-oxidative conditions in the barrels. In addition to the chemical oxidation of the phenolic acids, it is possible to have reactions between anthocyanins and free hydroxycinnamic acids to stabilize pigments, for example pinotin A or the reaction of ethanal and malvidin-3-glycoside yielding vitisin B9 can partly explain the decrease of anthocyanins and non-flavonoids [42]. Anthocyanins are not stable; breakdown reactions are possible but especially under the influence of oxygen during barrel aging protonated acetaldehyde reacts with different anthocyanins leading to polymerization reactions [41,43]. These direct or ethanal mediated condensation reactions between anthocyanin and tannin lead to the recorded increase in total and polymerized pigments [8,10,42]. The positive effect of barrel maturation on the formation of polymeric pigments is known [44,45] and oxygen availability and the anthocyanin to flavanol ratio were identified as regulating factors [5]. As previously mentioned, and contrary to what would be expected, no higher total anthocyanins concentration was found in the bottle matured wine in comparison with the barrel-aged wine (Table 2). As aforementioned, anthocyanins are unstable and the high levels of a young wine cannot be kept, but will decline with further wine aging, this counts as well for the reductive bottle storage of the bottle matured wine.

Beside the desired formation of polymeric pigments, it is as well possible for anthocyanins to break up and not add to the total wine color any longer [5,45,46]. The degree of ionization of anthocyanins still present in monomeric form was not different between the bottle matured wine and the wine samples aged for 12 months in barrels. A similar situation was found for the calculation product of ionized anthocyanins. The pH has strong influence on the ionization of anthocyanins and could explain these results, as the bottle matured wine and the 12 months barrel-aged wine did not differ in their pH.

#### 3.1.3. Chromatic Characteristics

The values for color intensity and tonality were 20% and 6% higher, respectively, in the barrel matured wine compared to the bottle matured wine (Table 2). This is the logical consequence of the increase in polymeric pigments discussed above. The CIELab analysis unfolded a more specific view, expressing for the barrel matured wine more yellow and red notes, while the color of the bottle matured wine was less yellow and less red (Table 2). Likewise, the barrel-aged wine resulted darker than the bottle stored bottle matured wine. The recorded findings about color evolution of barrel matured wine are in general agreement with the literature [6,8,10,47]. The color related to the copigmentation phenomena was with about 51% the same for the barrel matured wine and the bottle matured wine (Table 2). A total of 51% can be seen as a high value, keeping in mind that the color of young wines is reported to be to 30–50% due to the copigmentation phenomena [13]. This finding allows the conclusion that the difference in color observed between the barrel matured wine and the bottle matured were the result of the formation of polymeric pigments and a further indication that the wine, despite its maturation, still showed some characteristics typical of young wines.

### 3.2. Barrel-to-Barrel Variation

#### 3.2.1. The Effect of the Cooperage

**Figure 2:**
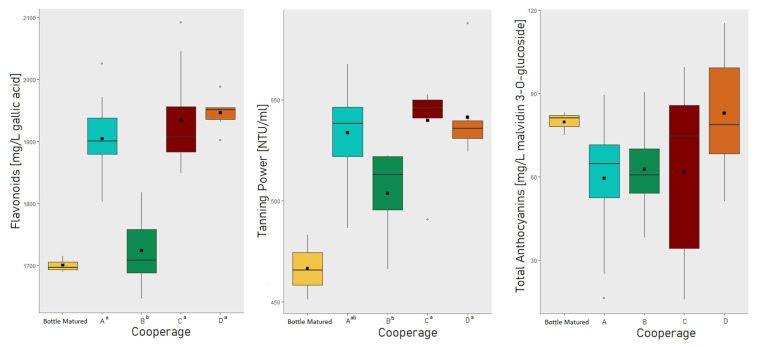
Boxplots of trial results for flavonoids, tanning power, and total anthocyanins with special separation for each cooperage and the bottle matured wine. For each parameter, cooperage codes followed by the same letter indicate not significantly different results at the 0.05 * level of significance.

The PCA (Figure 1) revealed overlapping areas for all cooperages. It is, therefore, consistent that no significant differences were found in between the cooperages A, C, and D for almost all analyzed parameters (Table 1 and Table 2). However, cooperage B revealed, depending on the analytical parameter, significant differences to all other cooperages, but also to just one or two of the other cooperages. Figure 2 shows exemplary the boxplots for flavonoids, tanning power and total anthocyanin with special separation for each cooperage and the bottle matured wine, while boxplots of wine color, phenols, and pigments are further available with Figures S1 and S2 in Supplementary Materials. In the boxplots the overlapping of cooperage A, C, and D becomes visible as well as the shift of cooperage B. However, no pattern could be identified for cooperage B, as for wine phenolics and chromatic characteristics, cooperage B differed in most cases to the other cooperages by a shift towards the bottle matured wine. For the general physical–chemical parameters however, the opposite is the case (Table 1). The explanations why cooperage B showed slightly different characteristics might have origin in a smaller oxygen uptake through the wood and rifts between the staves [19,21,48]. Therefore, this might be related to the cooperage’s production techniques and their choice of oak wood. To conclude, the wine aged for 12 months in different barrels varied in its phenolic and chromatic characteristics, but the cooperage of the individual barrel could not explain these variations. Furthermore, it was checked if the cooperage had an influence on the barrel-to-barrel variation by comparing the average coefficient of variation of the barrel-to-barrel variation of each cooperage (Table 3, following group calculations not displayed in the table). The choice of analytical parameter is critical (Table 3). The general physical–chemical parameters (Table 1 and Table 3) position at an average variation of 2.1% (sd. 0.5%), while the residing parameters are divided into three groups, anthocyanin (30% (sd. 7.9%)) and pigment (7.4% (sd. 3.1%)) related parameters and color plus residing phenolic parameters (4.7% variation, sd. 1.2% with the exception of flavanol monomers and proanthocyanidins fractions due to their observed high analytical method variation of on average 19.6%, sd. 4.7%). It can be concluded that the cooperages do not differentiate from each other with practical relevance in their internal variation for most parameters analyzed in this trial, exceptions are pigments and especially anthocyanin related parameters. 

#### 3.2.2. The Effect of the Barrel

The chemical characteristics analyzed in this experiment showed individual barrel-to-barrel variation for each cooperage with a range from 0.01% to 53.8% (Table 3). General physical–chemical parameters showed the lowest barrel-to-barrel variation in the trial (always < 2%). The exceptions were volatile acidity (7.9%, cooperage D) and residual sugar (12.6%, cooperage A); however, this variation is likely to originate in different microbiological activity and is not necessarily linked to the barrel characteristics. Most probably these results are explained by slight differences in temperature during the maturation period, different strains dominating the microbial population or a difference in the total number of active cells. The chromatic characteristics appeared in a range of 0.9% to 4.7% barrel-to-barrel variation, except for b* (18.1%, cooperage B) and angle of HUE (16.1%, cooperage B). Wine phenolics differences were found against total phenols/flavonoids (3.2%, average CV), non-flavonoids (5.1%, average CV), to total pigments (8.3%, average CV), polymerization index (8.5%, average CV) and finally ionized anthocyanins (25%, average CV) and total anthocyanins (37.2%, average CV) of barrel-to-barrel variation. It can be concluded that the effect of barrel aging on general characteristics such as density, alcoholic strength or total dry matter is either small or similar within the individual barrels [1,49,50]. The same is true for chromatic characteristics to a certain degree. The change from blue to yellow notes on the other hand was prone to a higher variation which is likely to be related to the variation found for anthocyanins. The observed variation for total pigments and the polymerization index led to the conclusion that polymerization reactions are probably influenced by the barrel, most likely by a variance in the permeation of oxygen. In comparison to the findings of Towey et al. [23], where volatile phenols were analyzed and a variation of 15% to 40% was identified, the variation for wine phenolics and color were generally lower. To check for the analytical method variation in the present trial, the coefficient of variation was calculated out of the triplet samples done for each measurement (Table 3). Regarding tannin fractions, it was not possible to discriminate wines, probably because of the observed analytical method variation in the present trial. 

In summary these findings indicate that the effect of barrel-to-barrel variation on chemical parameters of a red wine depend on each specific parameter and is not uniform. Especially the anthocyanins content, shows high variation between barrels in general and is to a lower degree as well impacted by the cooperage (Table 3 and Figure 2).

### 3.3. Required Barrel Number

The high number of barrel samples allowed for a backwards calculation based on the observed variation, to explain the relationship between precision and the required number of analytical samples. The relationship is that the higher the variation, the more samples of a batch are needed to display in their average the true population mean. Table 3 displays the required barrel number for each cooperage separately at 5%, 10% and 15% of variation around the true population mean. The average required sample number has been calculated for results with a precision of 2% to 20% around the true population mean (Table S1 in Supplementary Materials). The aim was to draw the link between the analytical parameter, the desired or available barrel sample number and the resulting precision of results. A precision of 10% for example means all results will be inside a range of 5% above and 5% below the true population mean. The results revealed that all phenolic and chromatic characteristics, except for the tannin fractions analysis and anthocyanin-related parameters, can be analyzed with only two barrel samples per barrel lot at a precision of 20%. This stands in contrast to on average seven barrels per barrel lot and a precision of 20% reported for the analysis of volatile phenols [23]. When increasing the precision more analytical parameters require higher sample numbers per barrel lot. At a 10% range around the true population mean, several analytical parameters require more than two barrels per lot, as for example total pigments and polymerization index (Table 3). Towey et al. [23], reported 27 barrels, on average, for the determination of volatile phenols in a 10% range around the true population mean. At 5% range around the true population mean, only clarity, tonality, and color due to copigmentation, as well as most physical–chemical parameters can be analyzed with up to two barrels per lot (Table 3). Non-flavonoids illustrate well the connection of variation and required sample size when comparing cooperage A and D with cooperage B and C (Table 3).

It can be summarized that the analysis of phenolic and chromatic characteristics requires less samples per barrel lot than the analysis of volatile phenols to achieve the same precision in their results. General physical–chemical parameters required the least samples due to a low barrel-to-barrel variation. To achieve reliable results in practical circumstances are for the analysis of general wine characteristics and wine color in most cases between one to three barrels per barrel lot sufficient (5% around the true population mean). Analytical parameters influenced by wine maturation, as the formation of polymeric pigments, polymerization of phenolics and especially anthocyanin related parameters require more samples per barrel lot, otherwise a reduction in the precision of the results needs to be accepted.

It is recommended that experimental designs including barrels take the expected variation of their analytical parameters into account. This can be done by choosing a reasonable sample size or by paying respect to it at the interpretation of the results. We would like to encourage others to calculate the variation from barrel to barrel in future trials, especially if a certain sample size is given. 

## 4. Conclusions

This study presents new insights into the maturation of wine during barrel aging, the phenolic composition of barrel-aged wine and the variation from cooperage to cooperage and from barrel to barrel. It could be shown in this trial that the influence of the individual barrels on the variation from barrel to barrel of wine phenolics and pigments was higher than the influence of the manufacturing cooperage. Chemical parameters analyzed in this study were prone to barrel-to-barrel variation at individual levels, overall ranging from almost zero up to 37% variation (average CV). Especially parameters related to anthocyanins were found to have a high barrel-to-barrel variation. The barrel-to-barrel variation of a chemical parameter influences the required sample size needed per analyzed batch. Detailed advise on the required sample size for certain chemical parameters at different levels of exactness were calculated and can be used as a help approaching measurements involving barrel lots, either in wine production or scientific trials. However, this experiment included only new barrels with the same toasting, while for barrel lots of different age and toasting level a qualified statement cannot be made. Due to the fading effect of the toasting a trial with old barrels could identify the variation fewer influenced by the barrel manufacturing and with stronger focus on the material wood, while on the other hand factors of material alteration during aging might play an additional role. The findings on barrel-to-barrel variation led to the recommendation for any future trials involving barrel lots to implement a variation analysis. To our best knowledge, this is the first study being disclosed with a high sample size to analyze the variating effect of barrels on the phenolic composition of wine. Therefore, more research on this field is required to extend and diversify the results of this trial. 

## Figures and Tables

**Table 1:** General physical–chemical parameters of red wines after 12 months of aging in oak barrels by cooperage and of a bottle matured sample, and cooperage effect with statistical difference between the cooperages.

Analytical Parameter	Cooperage Effect	Cooperage A	Cooperage B	Cooperage C	Cooperage D	Average (A, B, C, D)	Bottle Matured
Density (g/mL)	**	0.9917 ± 0.0001 ^ab^	0.9918 ± 0.0001 ^a^	0.9918 ± 0.0001 ^a^	0.9916 ± 0.0001 ^b^	0.9917 ± 0.0001	0.9909 ± 0.0000
Alcoholic Strength (% vol.)	**	13.4 ± 0.1 ^ab^	13.4 ± 0.0 ^ab^	13.3 ± 0.1 ^b^	13.4 ± 0.0 ^a^	13.4 ± 01	13.0 ± 0.0
Total Dry Matter (g/L)	**	29.7 ± 0.2 ^b^	29.9 ± 0.2 ^a^	29.7 ± 0.1 ^ab^	29.7 ± 0.1 ^b^	29.7 ± 0.2	27.3 ± 0.0
Residual Sugar (g/L)	***	1.8 ± 0.2 ^a^	1.5 ± 0.1 ^b^	2.0 ± 0.1 ^a^	1.9 ± 0.2 ^a^	1.8 ± 0.2	3.7 ± 0.1
Total Acidity (g tartaric acid/L)	***	5.67 ± 0.10 ^b^	5.91 ± 0.02 ^a^	5.65 ± 0.08 ^b^	5.63 ± 0.10 ^b^	5.71 ± 0.07	5.71 ± 0.00
Volatile Acidity (g acetic acid /L)	**	0.80 ± 0.05 ^ab^	0.73 ± 0.05 ^b^	0.83 ± 0.07 ^a^	0.75 ± 0.05 ^b^	0.78 ± 0.05	0.78 ± 0.00
Total SO_2_ (mg/L)	**	86 ± 1 ^a^	83 ± 1 ^b^	86 ± 1 ^a^	86 ± 1 ^a^	86 ± 1	88 ± 1
pH	ns	3.44 ± 0.01	3.45 ± 0.01	3.45 ± 0.02	3.44 ± 0.01	3.44 ± 0.01	3.44 ± 0.00
Ash (g/L)	**	3.30 ± 0.05 ^b^	3.37 ± 0.02 ^a^	3.29 ± 0.06 ^b^	3.28 ± 0.06 ^b^	3.31 ± 0.06	3.09 ± 0.03
Sulfates (mg/L)	ns	0.99 ± 0.01	0.98 ± 0.02	0.99 ± 0.01	0.99 ± 0.01	0.99 ± 0.01	0.38 ± 0.00
Chloride (mg/L)	***	0.065 ± 0.001 ^b^	0.069 ± 0.001 ^a^	0.0645 ± 0.001 ^b^	0.065 ± 0.001 ^b^	0.066 ± 0.001	0.069 ± 0.001

Results expressed as mean ± standard deviation. Means followed by the same letter in a line are not significantly different at the 0.01 ** or 0.001 *** level of significance; n.s. = without significant difference. Results for cooperages A, B, C, and D, are based on 20, 8, 9, and 12 barrels, respectively. Bottle matured results are based on 9 analytical replicates.

**Table 2:** Color, pigments, and phenolic composition of red wines after 12 months of aging in oak barrels by cooperage and of a bottle matured sample, and cooperage effect with statistical difference between the cooperages.

Analytical Parameter	Cooperage Effect	Cooperage A	Cooperage B	Cooperage C	Cooperage D	Average (A, B, C, D)	Bottle Matured
Total Phenols (mg/L gallic acid)	***	2050 ± 57 ^a^	1864 ± 65 ^b^	2081 ± 88 ^a^	2093 ± 33 ^a^	2022 ± 61	1948 ± 14
Flavonoids (mg/L gallic acid)	***	1904 ± 56 ^a^	1724 ± 66 ^b^	1934 ± 88 ^a^	1946 ± 26 ^a^	1877 ± 59	1701 ± 13
Non-flavonoids (mg/L gallic acid)	ns	145 ± 12	140 ± 3	147 ± 5	147 ± 9	145 ± 7	248 ± 1
Flavanol Monomers (mg/L)	ns	14 ± 3	15 ± 3	16 ± 2	19 ± 5	16 ± 3	12 ± 4
Oligomeric Proanthocyanidins (mg/L)	ns	64 ± 20	57 ± 11	63 ± 21	71 ± 20	64 ± 18	38 ± 13
Polymeric Proanthocyanidins (mg/L)	ns	438 ± 66	585 ± 128	512 ± 58	576 ± 66	528 ± 80	462 ± 60
Total Condensed Tannins (mg/L)	ns	516 ± 76	658 ± 141	591 ± 62	667 ± 91	608 ± 93	512 ± 49
Tanning Power (NTU/mL)	**	534 ± 24 ^ab^	504 ± 24 ^b^	540 ± 20 ^a^	541 ± 21 ^a^	530 ± 22	467 ± 16
Total Pigments (a.u.)	ns	15.1 ± 0.9	14 ± 2	15 ± 1	16.4 ± 0.8	15 ± 1	12.8 ± 0.2
Polymerization Index (%)	ns	54 ± 4	52 ± 3	54 ± 6	50 ± 4	52 ± 4	46 ± 1
Polymerized Pigments (a.u.)	**	8.1 ± 0.2 ^a^	7.4 ± 0.9 ^b^	8.1 ± 0.2 ^a^	8.2 ± 0.3 ^a^	8.0 ± 0.4	5.9 ± 0.0
Total Anthocyanins (mg/L malvidin 3-O-glucoside)	ns	59 ± 21	63 ± 20	62 ± 33	83 ± 24	67 ± 24	80 ± 4
Degree of Ionization of Anthocyanins (%)	ns	60 ± 17	60 ± 20	64 ± 22	50 ± 8	58 ± 17	57 ± 5
Ionized Anthocyanins (mg/L malvidin 3-O-glucoside)	ns	33 ± 7 ^a^	36 ± 12 ^a^	35 ± 10 ^a^	40 ± 6 ^a^	36 ± 9	45 ± 2
Color Intensity (a.u.)	***	20.03 ± 0.63 ^a^	18.71 ± 0.89 ^b^	20.22 ± 0.53 ^a^	20.48 ± 0.26 ^a^	19.86 ± 0.58	16.52 ± 0.16
Tonality	**	0.674 ± 0.007 ^a^	0.645 ± 0.02 ^b^	0.668 ± 0.012 ^ab^	0.671 ± 0.006 ^a^	0.664 ± 0.011	0.627 ± 0.004
Color due to Copigmentation (%)	ns	51 ± 2	51 ± 1	51 ± 2	51 ± 2	51 ± 1	52 ± 1
L*, Clarity (a.u.) [0 = black, 100 = colorless]	*	52.4 ± 0.8 ^ab^	53.8 ± 1.1 ^a^	52.0 ± 1.0 ^ab^	51.3 ± 0.7 ^b^	52.4 ± 0.9	55.7 ± 0.1
a*, Green-Red (a.u.) [green < 0 > red]	*	44 ± 1 ^ab^	42 ± 1 ^b^	44 ± 2 ^ab^	45 ± 1 ^a^	44 ± 1	41 ± 0.0
b*, Blue-Yellow (a.u.) [blue < 0 > yellow]	***	9.1 ± 1.1 ^a^	6.6 ± 1.2 ^b^	8.4 ± 0.9 ^ab^	9.6 ± 0.8 ^a^	8.5 ± 1.0	−1.2 ± 0.0
C*, Chroma (a.u.)	*	44.5 ± 1.3 ^ab^	42.8 ± 1.3 ^b^	44.7 ± 1.6 ^a^	45.8 ± 0.9 ^a^	44.5 ± 1.3	41.3 ± 0.0
H*, Tone or Angle of HUE (0–360°)	***	4.8 ± 0.6 ^b^	6.5 ± 1.0 ^a^	5.2 ± 0.5 ^b^	4.6 ± 0.4 ^b^	5.3 ± 0.7	−33.7 ± 2.5

Results expressed as mean ± standard deviation. Means followed by the same letter in a line are not significantly different at the 0.05 *, 0.01 ** or 0.001 *** level of significance; n.s. = without significant difference. Results for cooperages A, B, C, and D, are based on 20, 8, 9, and 12 barrels, respectively. Bottle matured results are based on 9 analytical replicates.

**Table 3:** Variation of general physical–chemical composition, color, pigments, and phenolic composition of red wines after 12 months of aging in oak barrels according to cooperage, and required barrel number per lot for different levels of precision.

Analytical Parameter	Analytical Method Variation (%)	Coefficient of Variation (CV) (%) and the Barrel Number Required for Results within the Called Percentage of the True Population Mean																
		Cooperage A				Cooperage B				Cooperage C				Cooperage D				Average CV
		CV	5%	10%	15%	CV	5%	10%	15%	CV	5%	10%	15%	CV	5%	10%	15%	
Density (g/mL)	0.1	0.0	1	1	1	0.0	1	1	1	0.0	1	1	1	0.0	1	1	1	0.0 ± 0.0
pH	0.1	0.4	1	1	1	0.4	1	1	1	0.3	1	1	1	0.5	1	1	1	0.4 ± 0.1
Alcoholic Strength (% vol.)	0.1	0.6	1	1	1	0.3	1	1	1	0.4	1	1	1	0.5	1	1	1	0.5 ± 0.1
Total Dry Matter (g/L)	0.1	0.6	1	1	1	0.3	1	1	1	0.6	1	1	1	0.4	1	1	1	0.5 ± 0.1
Sulfates (mg/L)	0.6	1.0	1	1	1	0.9	1	1	1	1.4	1	1	1	0.9	1	1	1	1 ± 0.2
Total SO_2_ (mg/L)	0.4	1.3	1	1	1	1.0	1	1	1	1.4	1	1	1	1.2	1	1	1	1.2 ± 0.2
Total Acidity (g tartaric acid/L)	0.2	1.7	1	1	1	1.8	1	1	1	0.3	1	1	1	1.4	2	1	1	1.3 ± 0.6
Ash (g/L)	0.9	1.5	1	1	1	1.8	1	1	1	0.7	2	1	1	1.9	2	1	1	1.5 ± 0.5
Chloride (mg/L)	0.3	1.8	2	1	1	1.8	1	1	1	0.8	1	1	1	1.8	2	1	1	1.5 ± 0.4
L*, Clarity (a.u.) [0 = black, 100 = colorless]	0.2	1.6	1	1	1	2.0	2	1	1	2.0	2	1	1	1.4	1	1	1	1.7 ± 0.3
Tonality	0.7	1.0	1	1	1	3.0	3	1	1	1.9	2	1	1	0.9	1	1	1	1.7 ± 0.9
Color due to Copigmentation (%)	1.1	3.6	5	2	1	0.6	1	1	1	3.2	4	1	1	3.3	4	1	1	2.7 ± 1.2
a*, Green-Red (a.u.) [green < 0 > red]	0.2	2.7	3	1	1	2.7	3	1	1	3.5	4	1	1	1.8	2	1	1	2.7 ± 0.6
C*, Chroma (a.u.)	0.2	2.9	3	1	1	3.0	3	1	1	3.6	5	2	1	1.9	2	1	1	2.9 ± 0.6
Color Intensity (a.u.)	0.5	3.2	4	1	1	4.7	8	2	1	2.6	3	1	1	1.2	1	1	1	2.9 ± 1.3
Total Phenols (mg/L gallic acid)	1.3	2.8	3	1	1	3.5	4	1	1	4.2	6	2	1	1.6	1	1	1	3 ± 1
Flavonoids (mg/L gallic acid)	1.3	3.0	3	1	1	3.8	5	2	1	4.5	7	2	1	1.4	1	1	1	3.2 ± 1.2
Tanning Power (NTU/mL)	2.7	4.4	7	2	1	4.7	7	2	1	3.7	5	2	1	3.9	5	2	1	4.2 ± 0.4
Non-flavonoids (mg/L gallic acid)	2.3	8.5	23	6	3	2.4	2	1	1	3.5	4	1	1	5.8	11	3	2	5.1 ± 2.3
Polymerized Pigments (a.u.)	0.9	2.9	3	1	1	12.3	48	12	6	2.6	3	1	1	3.7	5	2	1	5.3 ± 4
Volatile Acidity (g acetic acid/L)	0.2	6.1	12	3	2	6.7	14	4	2	6.5	20	5	3	7.9	15	4	2	6.8 ± 0.7
Polymerization Index (%)	2.9	7.6	19	5	3	6.6	14	4	2	11.8	44	11	5	8.0	21	6	3	8.5 ± 2
Residual Sugar (g/L)	2.5	12.6	51	13	6	8.7	6	2	1	4.0	17	5	2	7.3	24	6	3	8.2 ± 3.1
Total Pigments (a.u.)	3.6	5.9	11	3	2	13.2	55	14	7	9.5	29	8	4	4.7	7	2	1	8.3 ± 3.3
H*, Tone or Angle of HUE (0–360°)	0.7	12.8	52	13	6	16.1	104	26	12	10.4	34	9	4	8.4	23	6	3	11.9 ± 2.9
b*, Blue-Yellow (a.u.) [blue < 0 > yellow]	0.6	12.6	51	13	6	18.1	104	26	12	11.5	42	11	5	8.8	25	7	3	12.8 ± 3.4
Polymeric Proanthocyanidins (mg/L)	12.3	15.0	71	18	8	21.9	151	38	17	11.4	41	11	5	11.4	42	11	5	14.9 ± 4.3
Total Condensed Tannins (mg/L)	10.6	14.8	69	18	8	21.4	145	37	17	10.5	35	9	4	13.6	59	15	7	15.1 ± 4
Flavanol Monomers (mg/L)	13.8	20.0	125	32	14	22.5	160	40	18	12.9	53	14	6	27.0	229	58	26	20.6 ± 5.1
Ionized Anthocyanins (mg/L malvidin 3-O-glucoside)	17.4	21.4	144	36	16	33.5	353	89	40	29.4	271	68	31	15.8	79	20	9	25 ± 6.9
Degree of Ionization of Anthocyanins (%)	9.0	28.6	257	65	29	33.1	344	86	39	33.7	357	90	40	16.1	82	21	10	27.9 ± 7.1
Oligomeric Proanthocyanidins (mg/L)	15.4	30.9	310	76	34	19.2	116	29	13	33.7	356	89	40	28.3	252	63	28	28 ± 5.5
Total Anthocyanins (mg/L malvidin 3-O-glucoside)	15.9	35.6	398	100	45	30.9	299	75	34	53.8	908	227	101	28.35	253	64	29	37.2 ± 10

The analytical method variation was calculated with the coefficient of variation of each barrel sample triplicate and then averaged for the respective analytical parameter. Results for cooperages A, B, C, and D, are based on 20, 8, 9, and 12 barrels, respectively. The percentage (5%, 15% and 20%) is a range around the true barrel lot mean of the respective chemical parameter. The average result of a barrel lot analysis is predicted to be within the respective range, if the barrel lot contains of the required number of barrels or more. The barrel numbers were calculated at 95% confidence, 80% power and rounded up only.

## Data Availability

Data are contained within the article and Supplementary Materials.

## Supplementary Materials

The following are available online at https://www.mdpi.com/article/10.3390/foods10071669/s1, Table S1: Average required barrel number per lot for different levels of precision. Figure S1: color, pigments of red wines after 12 months of aging in oak barrels displayed in boxplots for cooperage A, B, C, and D, as well as the bottle matured wine. Figure S2: phenolic composition of red wines after 12 months of aging in oak barrels displayed in boxplots for cooperage A, B, C, and D, as well as the bottle matured wine.
