# Supplementary material for: Effect of Barrel-to-Barrel Variation on Color and Phenolic Composition of a Red Wine
Source: Foods. 2021 Jul 20;10(7):1669. doi: 10.3390/foods10071669 (PMC8303824; doi:10.3390/foods10071669)
Supplement: Supplementary file 1 [file foods-10-01669-s001.zip › foods-1255795-supplementary.pdf]

Table S1: Average required barrel number per lot for different levels of precision.

Analytical Parameter	Barrel number required for results within the called percentage of the true population mean				
	2%	5%	10%	15%	20%
Density (g/mL)	1	1	1	1	1
pH	1	1	1	1	1
Alcoholic Strength (% vol.)	1	1	1	1	1
Total Dry Matter (g/L)	1	1	1	1	1
Sulfates (mg/L)	3	1	1	1	1
Total SO_2 (mg/L)	3	1	1	1	1
Total Acidity (g tartaric acid/L)	5	1	1	1	1
Ash (g/L)	5	1	1	1	1
Chloride (mg/L)	5	1	1	1	1
L*, Clarity (a.u.) [0=black, 100=colourless]	7	1	1	1	1
Tonality	7	2	1	1	1
Colour due to Copigmentation (%)	12	2	1	1	1
a*, Green-Red (a.u.) [green < 0 > red]	15	3	1	1	1
C*, Chroma (a.u.)	17	3	1	1	1
Colour Intensity (a.u.)	19	3	1	1	1
Total Phenols (mg/L gallic acid)	19	4	1	1	1
Flavonoids (mg/L gallic acid)	22	4	1	1	1
Tanning Power (NTU/mL)	35	6	2	1	1
Non-flavonoids (mg/L gallic acid)	61	10	3	2	1
Polymerised Pigments (a.u.)	88	14	4	2	1
Volatile Acidity (g acetic acid/L)	92	15	4	2	1
Polymerisation Index (%)	145	24	6	3	2
Residual Sugar (g/L)	150	24	6	3	2
Total Pigments (a.u.)	151	25	7	3	2
H*, Tone or Angle of HUE (0-360°)	191	31	8	4	2
b*, Blue-Yellow (a.u.) [blue < 0 > yellow]	232	38	10	5	3
Polymeric Proanthocyanidins (mg/L)	474	76	19	9	5
Total Condensed Tannins (mg/L)	477	77	20	9	5
Flavanol Monomers (mg/L)	883	142	36	16	9
Ionised Anthocyanins (mg/L malvidin 3-O-glucoside)	1291	207	52	23	13
Degree of Ionisation of Anthocyanins (%)	1453	233	59	26	15
Oligomeric Proanthocyanidins (mg/L)	1599	256	64	29	16

Total Anthocyanins (mg/L malvidin 3-O-glucoside)	2845	456	114	51	29

The percentage (2%, 5%, 15% and 20%) is a range around the true barrel lot mean of the respective chemical parameter. The average result of a barrel lot analysis is predicted to be within the respective range, if the barrel lot contains of the required number of barrels or more. Results were calculated at 95% confidence, 80% power first for the cooperages A, B, C and D, which were based on 20, 8, 9 and 12 barrels, respectively. The required barrel numbers were averaged between the cooperages and rounded up only.

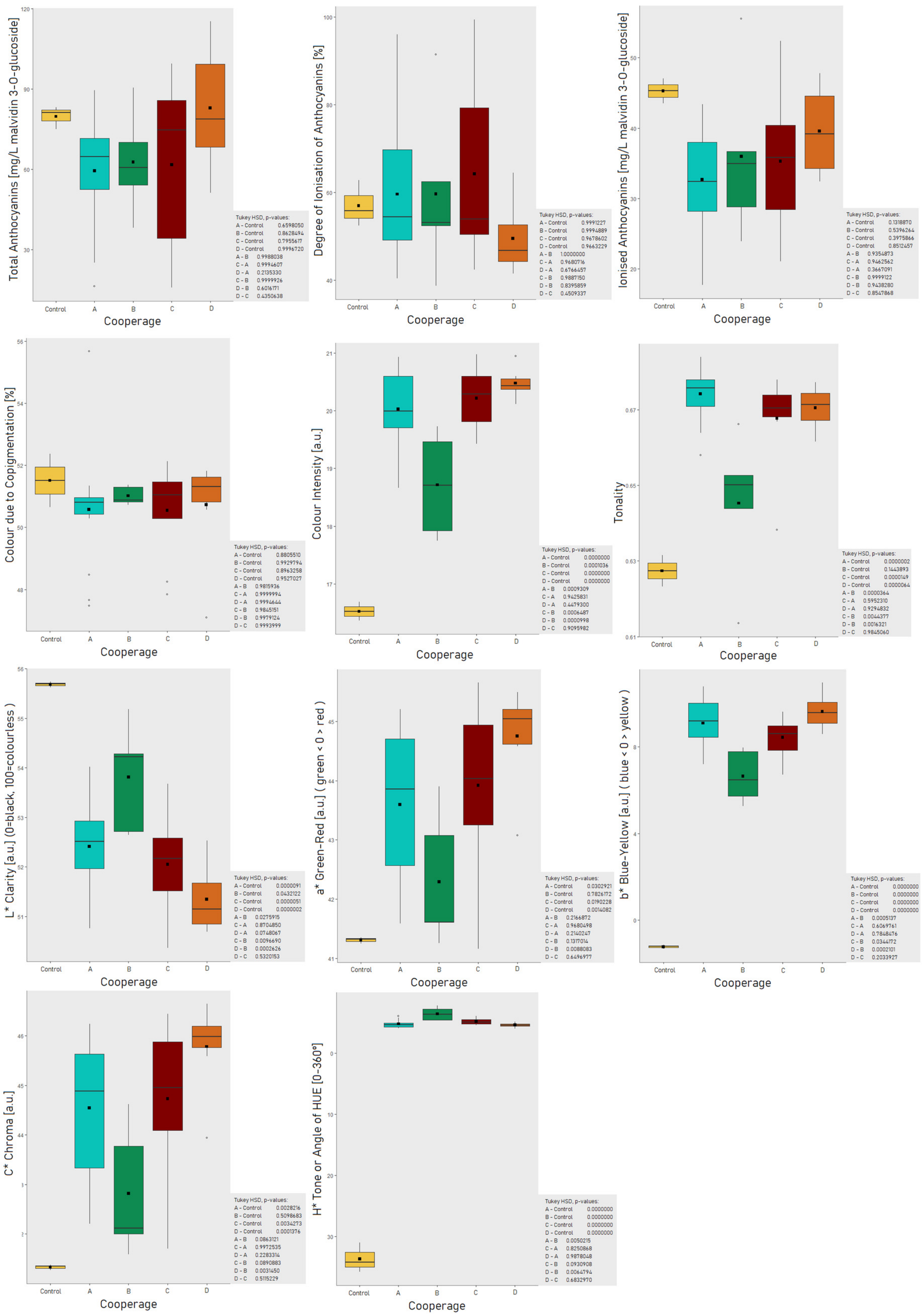

Figure S1: color, pigments of red wines after 12 months of aging in oak barrels displayed in boxplots for cooperage A, B, C, and D, as well as the bottle matured wine

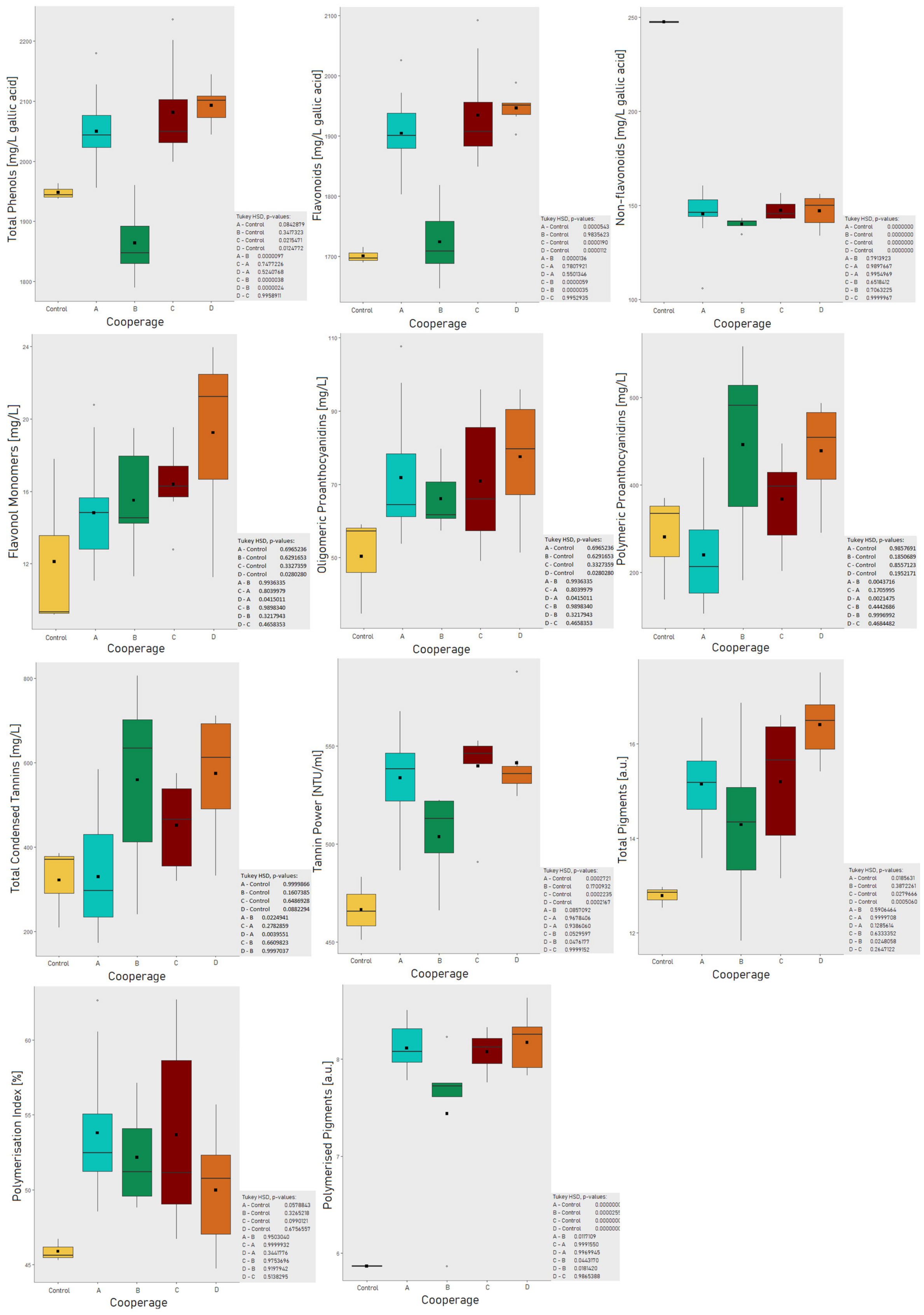

Figure S2: phenolic composition of red wines after 12 months of aging in oak barrels displayed in boxplots for cooperage A, B, C, and D, as well as the bottle matured wine.
